# 3D Printing for Bio-Synthetic Biliary Stents

**DOI:** 10.3390/bioengineering6010016

**Published:** 2019-02-09

**Authors:** Christen J. Boyer, Moheb Boktor, Hrishikesh Samant, Luke A. White, Yuping Wang, David H. Ballard, Robert C. Huebert, Jennifer E. Woerner, Ghali E. Ghali, Jonathan S. Alexander

**Affiliations:** 1 Molecular and Cellular Physiology, Health Sciences Center, Louisiana State University, Shreveport, LA 71103, USA; cboye2@lsuhsc.edu (C.J.B.); lwhit9@lsuhsc.edu (L.A.W.); 2 Oral and Maxillofacial Surgery, Health Sciences Center, Louisiana State University, Shreveport, LA 71103, USA; jwoern@lsuhsc.edu (J.E.W.); GGhali@lsuhsc.edu (G.E.G.); 3 Gastroenterology and Hepatology, Health Sciences Center, Louisiana State University, Shreveport, LA 71103, USA; mbokto@lsuhsc.edu (M.B.); hsaman@lsuhsc.edu (H.S.); 4 Obstetrics and Gynecology, Health Sciences Center, Louisiana State University, Shreveport, LA 71103, USA; YWang1@lsuhsc.edu; 5 Mallinckrodt Institute of Radiology, School of Medicine, Washington University, St. Louis, MO 63110, USA; davidballard@wustl.edu; 6 Gastroenterology and Hepatology, Mayo Clinic, Rochester, MN 55905, USA; Huebert.Robert@mayo.edu

**Keywords:** 3D printing, hepatobiliary stent, tissue engineering, medical device, stem cells, personalized medicine

## Abstract

Three-dimensional (3D) printing is an additive manufacturing method that holds great potential in a variety of future patient-specific medical technologies. This project validated a novel crosslinked polyvinyl alcohol (XL-PVA) 3D printed stent infused with collagen, human placental mesenchymal stem cells (PMSCs), and cholangiocytes. The biofabrication method in the present study examined 3D printing and collagen injection molding for rapid prototyping of customized living biliary stents with clinical applications in the setting of malignant and benign bile duct obstructions. XL-PVA stents showed hydrophilic swelling and addition of radiocontrast to the stent matrix improved radiographic opacity. Collagen loaded with PMSCs contracted tightly around hydrophilic stents and dense choloangiocyte coatings were verified through histology and fluorescence microscopy. It is anticipated that design elements used in these stents may enable appropriate stent placement, provide protection of the stent-stem cell matrix against bile constituents, and potentially limit biofilm development. Overall, this approach may allow physicians to create personalized bio-integrating stents for use in biliary procedures and lays a foundation for new patient-specific stent fabrication techniques.

## 1. Introduction

Biliary obstruction can be caused by both benign and malignant conditions including iatrogenic bile duct injury and chronic pancreatitis as well as cholangiocarcinoma and pancreatic cancer [1,2]. Relief from such obstructions can help minimize obstructive jaundice and reduce the risk of cholangitis [3,4]. The endoscopic placement of hepatobiliary stents was first performed in 1980 to restore biliary flow [5,6]. Since then, such endoscopic techniques have become favored alternatives to surgery due to their less invasive nature and decreased morbidity associated with these procedures [7].

Several types of stents have been developed over the years, each with their own advantages and disadvantages. Plastic stents were used first and today are still the most commonly used device [5,6]. In the late 1980s, self-expanding metal stents were first used in the biliary tract [8,9,10]. Compared to plastic stents, self-expanding metal stents have decreased mortality as well as the number of re-interventions, however, self-expanding metal stents are more expensive [4].

More recently, drug-eluting stents have been developed as an approach to maintain stent patency [11]. Both plastic and metal stents with anti-reflux valves have been explored as a means to decrease cholangitis and increase patency [12]. The use of biodegradable biliary stents has also been explored. In many clinical scenarios, biliary stents are only needed temporarily [13,14]. A biodegradable stent that dissolves allowing its remnants to be expelled into the duodenum would eliminate the need for a secondary device removal procedure, which would increase the risk for recurrent biliary occlusion or stenosis.

Despite many advances in stent development, one major problem that remains is the progressive loss of stent patency over time. Several factors contribute to this loss of patency. It is widely accepted that biofilm formation on the stent is a common cause of stent failure. Bacteria gain access to biliary stents by migrating from either the portal venous system or through the sphincter of Oddi [15]. Microorganisms may then adhere to the stent and begin to proliferate and secrete exopolysaccharides to form biofilms that resist antibiotic treatments. Biofilm creation, in turn, is thought to contribute to biliary ‘sludge’ formation that ultimately leads to the loss of stent patency [16].

One area that remains unexplored is the development and use of 3D printed hepatobiliary stents. 3D printing is an emerging technology in medicine, and it would allow for the rapid production of custom medical products that are relatively inexpensive [17]. The application of 3D printed stents has been explored for use in many diseases. Several examples include the use of 3D printed vascular stents for percutaneous coronary intervention, airway stents for the treatment of central airway obstruction, esophageal stents for palliative care in inoperable esophageal malignancies, and ureteral stents which facilitate urine drainage from the kidney [18,19,20,21].

Cytocompatible, hydrophilic, and drug-deliverable 3D printed materials and techniques have been recently explored and hold great promise in cell culture models, medical devices, and tissue-engineered substrates [22,23,24,25]. Such materials include 3D printed polyvinyl alcohol (PVA), a water soluble polymer. PVA filaments demonstrate favorable 3D printability using fused deposition modeling (FDM) techniques which can be post-print crosslinked (XL) to form stable 3D hydrogels [26]. The 3D printed crosslinked polyvinyl alcohol (XL-PVA) method is capable of binding biomolecules, drugs, and cells of interest due to the molecular complexes formed during crosslinking. Such materials have applications in a variety of medical devices and wound care substrates. In this study, the XL-PVA 3D printing method was coupled with collagen injection molding to create hepatobiliary stents which have been engineered to support engraftment with human placental stem cells and cholangiocytes.

## 2. Materials and Methods

### 2.1. PVA Stent Fabrication and Crosslinking

Porous tubular stents (25 mm length, 5 mm external diameter, and 3 mm internal diameter) were created with free computer aided design (CAD) software (Tinker-CAD, AutoDesk, San Francisco, CA, USA). 96 square pores (1 mm × 1 mm) were created in the stent design with 1 mm spacing between each pore. PVA filaments (AquaSolve™, Formfutura, Nijmegen, Netherlands, 1.75 mm diameter) were 3D printed into the designed stent pattern at 201 °C using a consumer grade 3D printed (MakerBot Replicator desktop 3D printer, MakerBot Industries LLC, Brooklyn, NY, USA) with supports turned off and raft turned on. PVA 3D printed stents were immersed in distilled water briefly to fuse layers, and cross-linked (XL) by placing the stents in a gas vapor desiccator with two separate containers containing 20 mL of 6.25% glutaraldehyde (GA) (EMD Millipore Corporation, Darmstadt, Germany) and 10 mL of concentrated hydrochloric acid (HCl) (Fisher Scientific, Hampton, NH, USA) at 42 °C for 24 h. XL-PVA stents were next rinsed extensively in distilled water and soaked in 70% ethanol for 24 h. XL-PVA stents were rinsed again and placed in 1X phosphate buffer solution (PBS) for storage.

### 2.2. Collagen Injection Mold Chamber Fabrication

Cylindrical injection mold chambers and stent lumen maturation plugs were designed with free CAD software (Tinker-CAD) and 3D printed on a consumer grade stereolithography 3D printer (Formlabs, Form2, Somerville, MA, USA) using flexible resin (Formlabs) with supports turned on. 3D printed parts were rinsed in 70% isopropyl alcohol (Form Wash, Formlabs) and then cured at 60 °C with shortwave ultraviolet light (UV) for 1 h (Form Cure, Formlabs). Injection mold chambers and maturation plugs were next rinsed in 70% ethanol followed by a 1X PBS wash in a sterile tissue culture hood and stored there until used in experiments.

### 2.3. Barium Sulfate Coating and X-Ray Imaging

Polycaprolactone (PCL) (Sigma Aldrich, St. Louis, MO, USA) (molecular weight 80,000) and E-Z-HD™ barium sulfate (BS) (E-Z-EM Canada Inc., Quebec, Canada) were mixed in chloroform (CF) (Sigma Aldrich) to form 10% PCL:10% BS:80% CF mixture. Hydrated XL-PVA stents were dipped in the PCL-BS-CF mixtures on each tip’s end and the chloroform was allowed to evaporate before placing the stents back into 1X PBS. Final dry PCL-BS coatings on stent tips contained 50%PCL:50%BS. XL-PVA stents and versions with PCL-BS coatings were imaged with an OEC 9900 Elite C-Arm System X-ray (General Electric, Fairfield, CT, USA) in 10 mL of 1X PBS.

### 2.4. XL-PVA Swelling Analysis

For swelling studies, XL-PVA pieces (n = 5) were hydrated overnight in 1X PBS and dehydrated. Each hydrated and dehydrated form was weighed to obtain average weights. To calculate mass change percentage (ΔM%), the average hydrated weight (M1) was subtracted from the average dry weight (M2), then divided by the hydrated weight (M1) and multiplied by 100. The mass swelling ratio was calculated by taking the ratio between the average mass of the hydrated XL-PVA (M1) and the average mass of the dehydrated XL-PVA (M2), where M1 is divided by M2. The results were statistically analyzed by using Student’s *t*-test and the data were expressed as the mean ± standard deviation (SD). A *p* value of <0.05 was considered statistically significant.

### 2.5. Collagen Preparation

Rat tail type 1 collagen matrices were prepared by a modification of the protocol previously published by Benoit et al. [27]. Briefly, rat tail tendons were manually excised, washed with 100% isopropanol (Thermo Fisher Scientific, Waltham, MA, USA) and dissolved in sterile 4 mM acetic acid (Sigma Aldrich) for 24 h at 4 °C under constant agitation. Collagen solution was filtered through a 250 μm nylon filter (Spectrum Labs, Rancho Dominguez, CA, USA), centrifuged at 19× *g* for 20 min at 4 °C and snap frozen. Using a bench-top manifold freeze-dryer (Millrock Technology, Kingston, NY, USA), frozen aliquots were dried and stored at −20 °C for future use. Twenty-four hours prior to experiments, freeze-dried collagen was resolubilized in cold 0.012 M hydrochloric acid (HCl) (Sigma Aldrich) at 2.5 mg/mL^−1^ final collagen concentration and incubated overnight at 4 °C with gentle agitation. On the day of the experiment, 0.8 mL of cold 5X PBS was added to 3.2 mL of dissolved collagen gel and the pH was titrated with 0.5 M sodium hydroxide (NaOH) (Sigma Aldrich) to 7.4.

### 2.6. Isolation of Mesenchymal Stem Cells

Human placentas were collected from normal term pregnancies. Collection of human placenta for mesenchymal stem cell isolation was approved by the Institutional Review Board (IRB) (approval number CR00001345_STUDY00000614). Human placental mesenchymal stem cells (PMSCs) were obtained by cultivation of microvilli after elimination of villous trophoblasts. The villous tissue was dissected from different cotyledons, excluding chorionic and basal plates. After rinsing with ice-cold PBS, the villous tissue was next digested with trypsin (0.125% trypsin solution (Sigma Aldrich) containing 0.1 mg/mL DNase I and 5 (Sigma Aldrich) mM MgCl_2_ (Sigma Aldrich) in Dulbecco’s modified Eagle’s medium (DMEM) (Sigma Aldrich) at 37 °C for 90 min. The isolated PMSCs were incubated with DMEM supplemented with 10% fetal bovine serum (FBS) (Atlanta Biological, Flowery Branch, GA, USA) and 1% penicillin/streptomycin (P/S) (Sigma Aldrich) at 37 °C with 5% CO_2_. PMSCs were characterized by positive expression of mesenchymal stem cell cluster of differentiation (CD) markers CD73, CD90, and negative expression of CD34 and Human Leukocyte Antigen–antigen D Related (HLA-DR) (BD Pharmingen, Franklin Lakes, NJ, USA). PMSCs were monitored using flow cytometry (BD LSR II Flow Cytometer, BD Biosciences, San Jose, CA, USA) and immunofluorescent staining for octamer binding transcription factor 4 (Oct4), CD133 (Santa Cruz, San Diego, CA, USA) and CD44 (Abcam, Cambridge, MA, USA).

### 2.7. Stem Cell Collagen Injection Molding and Stent Maturation

All cell culture was performed under standard aseptic techniques to reduce contamination. PMSC (passage 9) was grown to confluency in 75 cm^2^ flat bottom flasks at 37 °C with 7.5% carbon dioxide (CO_2_), and 100% humidity. Cells were washed with PBS/ethylenediaminetetraacetic acid (PBS-EDTA) (Sigma Aldrich), trypsinized (Trypsin solution from porcine pancreas, Sigma Aldrich), collected through centrifugation, and re-suspended in DMEM (media) containing 10% FBS, (Atlas Biologicals, Fort Collins, CO, USA), 1% P/S (Sigma Aldrich), and 4.5 g of glucose/liter. Resuspended PMSCs were mixed with the prepared dissolved collagen solution to form a 12 mL mixture (8 mL of cell suspension in 4 mL of collagen gel solution). PMSC/collagen was seeded in the 3D printed injection molding chambers containing XL-PVA stents and maturation plugs. 2 mL of the PMSC-collagen mixture was added to each injection molding chamber and the total cell count for each chamber was 200,000 cells per chamber. The loaded injection molding chambers tissue were next incubated at 37 °C for 1 h., to polymerize the collagen around the 3D prints. Next, maturation plugs containing XL-PVA stents and polymerized collagen/PMSC were removed from each injection molding chamber and placed in 25 cm^2^ tissue culture flask with 25 mL of media. Maturation plugs were removed at day 5 and PMSC/collagen stents were allowed to mature for a total of 7 days.

Additional 48-well tissue culture plates were seeded with the PMSC-collagen mixtures and pure collagen as a control. Each well was seeded with 0.5 mL of the solutions and PMSC versions contained 50,000 cells per well. The loaded 48 well tissue culture plates were next incubated at 37 °C for 1 h., to polymerize the collagen in the wells. Contraction assays measuring the diameter of the gel were performed according to established protocols [27]. Collagen gels in the 48-well plates were detached around the edges with a glass pipet tip and 0.5 mL of media was added to each well. Collagen contraction was monitored for 1 week, and images were taken periodically on days 0, 3, 5, and 7), and analyzed with ImageJ software. The results were statistically analyzed by using Student’s *t*-test, and the data were expressed as the mean ± standard deviation (SD). A *p* value of <0.05 was considered statistically significant.

### 2.8. Cholangiocyte Seeding

Human primary cholangiocytes (Celprogen, Torrance, CA, USA) were grown to confluency in 75 cm^2^ tissue culture flask with human cholangiocyte primary cell culture complete growth media with serum and antibiotics (Celprogen). The obtained primary cholangiocytes were stated to have positive markers for cytokeratin (CK) CK7, CK19, glutamyl transpeptidase, aquaporin −1 (Aqp1), oval cell markers (OV) OV6 and OV1, and epithelial specific antigen (ESA). Cells were washed with PBS-EDTA, trypsinized, collected through centrifugation, and re-suspended in Celprogen media. Collagen injection molded XL-PVA stents were each mixed with 5 mL of cholangiocyte media containing 663,000 cell count and allowed to incubate overnight. Stents were next transferred to new 25 cm^2^ flask and 25 mL of fresh Celprogen media was added. Stents were allowed to mature for an additional week. Final cell-laden stents were analyzed live and fixed in 10% formalin (Thermo Fisher) for imaging and histology processing. For histology, fixed stents were embedded in parafilm wax, sectioned, and stained with hematoxylin and eosin (H&E) (Sigma Aldrich) for viewing.

### 2.9. Imaging

3D printed XL-PVA and collagen injection molded versions seeded with cells were characterized with a HITACHI 4800 high resolution scanning electron microscope with Gatan Cryo features (Cryo-SEM). Cell cultures and external and internal cell layers of bioengineered stents were also monitored through phase and fluorescence imaging with Hoechst 33342 (Thermo Fisher) on an Evos FL cell imaging system (Thermo Fisher). H&E stained sections were viewed with an EVOS XL Core imaging system (Thermo Fisher).

## 3. Results

### 3.1. Fabrication of XL-PVA Stents and Collagen Injection Molding Chamber

**Figure 1:**
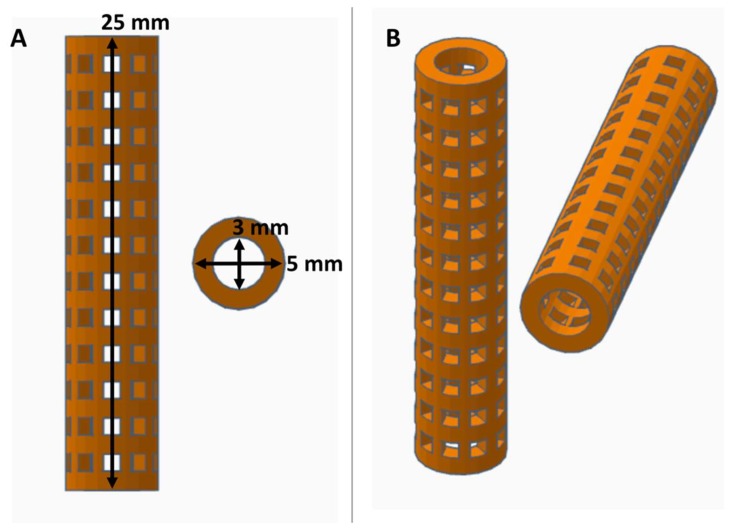
Views of computer aided design (CAD) stent with dimensions displayed in orthographic view (**A**) and in perspective view (**B**).

**Figure 2:**
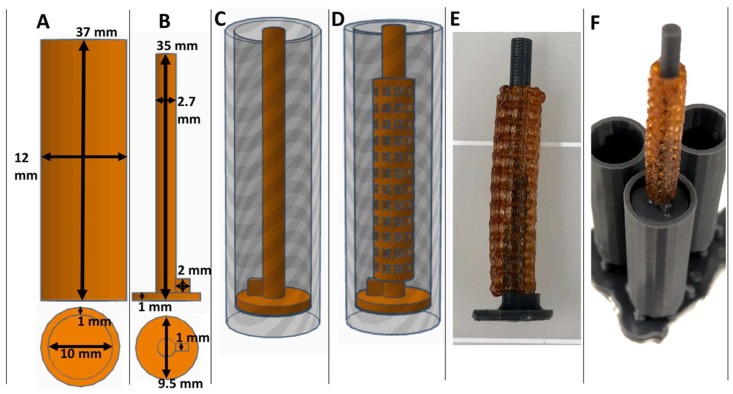
Views of CAD collagen injection molding chamber (**A**) and maturation plug (**B**) with dimensions displayed. CAD collagen injection molding chamber with maturation plug inserted (**C**) and with stent placement (**D**). Images of 3D printed actual crosslinked polyvinyl alcohol (XL-PVA) stents, over a maturation plug (**E**) and with the injection molding chambers (**F**).

**Figure 3:**
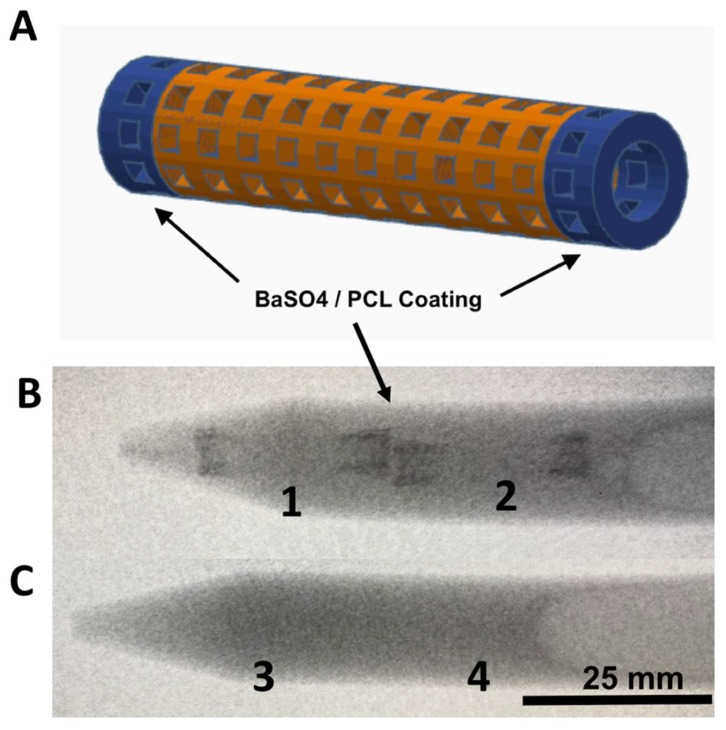
Images of CAD stent in (**A**), X-ray images of 3D printed XL-PVA stents with barium coated tips (**B**; 1 and 2), and X-ray image of control XL-PVA stents (**C**; 3 and 4). Black arrows point to regions of interest containing barium sulfate (scale bar = 25 mm).

Consumer grade free software and a MakerBot 3D printer were capable of forming reproducible stent structures from PVA filaments printed at 201 °C. (see Figure 1 and Figure 2). 3D printed PVA stents were successfully cross-linked with the HCl/GA gas crosslinking method previously described (see Figure 2D,E). The Form2 SLA 3D printer was successful at fabricating collagen injection molding chambers and maturation plugs (see Figure 2). The injection molding chambers and maturation plugs allowed for smooth integration with each part including the XL-PVA stents (see Figure 2D,E). XL-PVA stents coated with PCL/BA showed improved visibility under x-ray imaging when compared to control XL-PVA stents in PBS (see Figure 3).

### 3.2. XL-PVA Swelling Analysis

**Figure 4:**
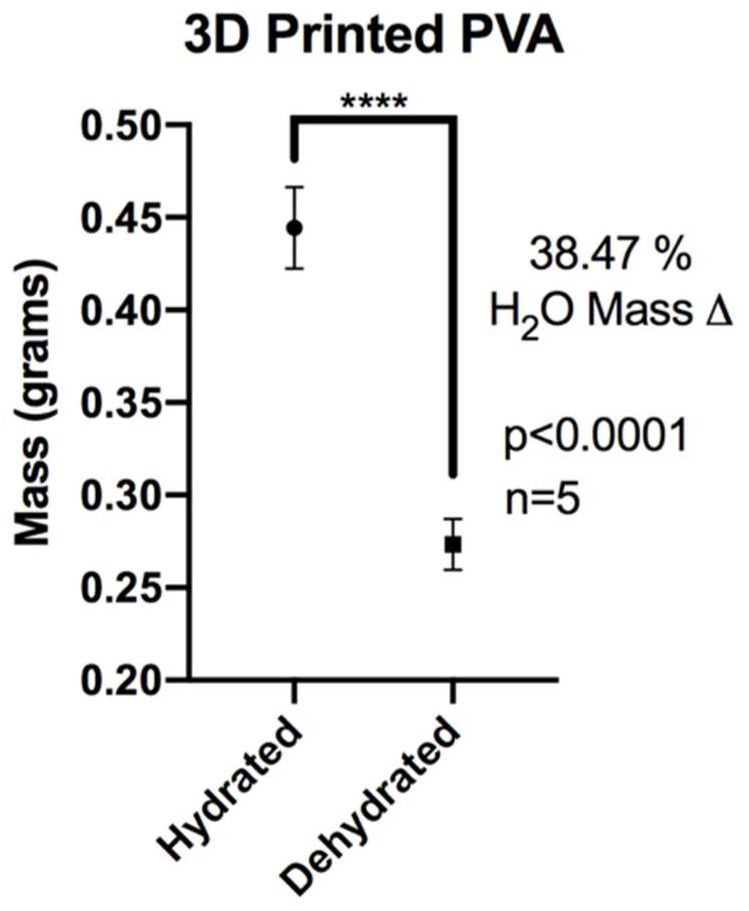
Chart displaying average masses of XL-PVA in hydrated and dehydrated forms. (**** = *p* < 0.0001).

Hydrated samples of XL-PVA (n = 5) had a mean mass (grams) ± standard deviation of 0.4444 ± 0.0220. Dehydrated samples of same XL-PVA samples (n = 5) had a mean ± standard deviation mass (grams) of 0.2734 ± 0.0137. For hydrated versus dehydrated XL-PVA, a significant difference was found for the mass totals (*p* < 0.0001). The change in mass percentage from hydrated to dehydrated XL-PVA was calculated as 38.47% (see Figure 4). The mass swelling ratio for hydrated and dehydrated XL-PVA was calculated as 1.625.

### 3.3. Hybrid Stent Cell Culture and Imaging

**Figure 5:**
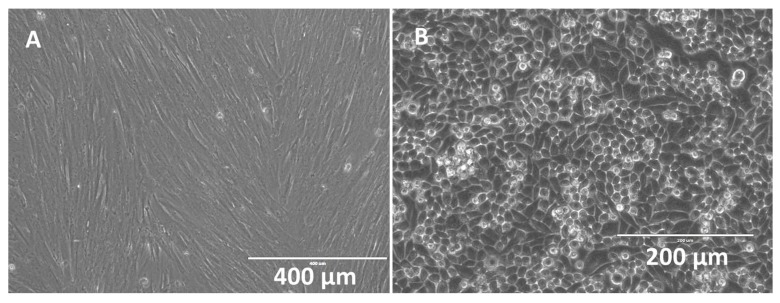
Phase microscopy images of confluent human placental mesenchymal stem cells (PMSCs) (**A**) (scale bar = 400 μm) and cholangiocytes (**B**) (scale bar = 200 μm).

Human PMSCs and cholangiocytes were successfully cultured on stents; these displayed unique morphologies when compared under phase microscopy. PMSCs displayed typical elongated and uniform web-like patterns while the cholangiocytes displayed typical circular and cobblestone patterns (see Figure 5). Under flow cytometry, PMSCs showed positive (+) expression for several markers (CD73+, CD90+) and negative (−) for markers CD34− and HLADR−. Under immunofluorescent imaging PMSCs showed CD133+, CD44+, and Oct4+.

**Figure 6:**
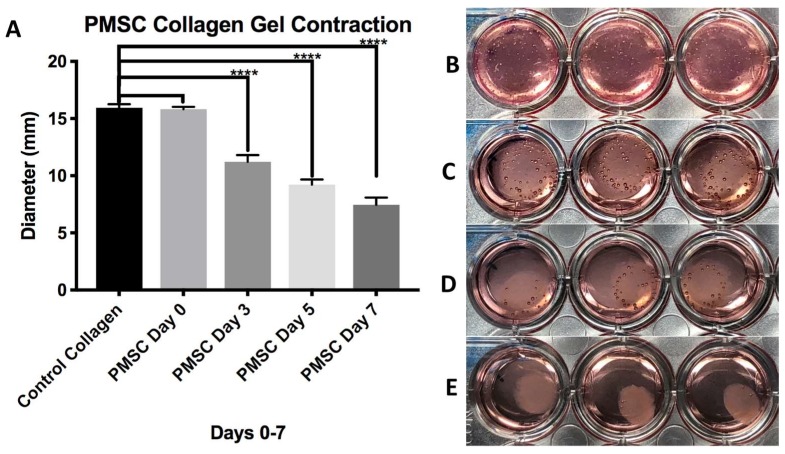
Chart displaying average diameter of control collagen gels and PMSC seeded collagen gels at days 0–7 days (**A**). Images on right show collagen gels seed with PMSCs at day 0 (**B**), day 3 (**C**), day 5 (**D**), and day 7 (**E**). (**** = *p* < 0.0001).

Collagen gels loaded with PMSCs showed significant contraction in 48-well cell culture plates and around the 3D printed XL-PVA stents. In 48-well plates, the diameters of the gels were measured and images were taken at the start of the experiment day 0 and at days 3, 5, and 7. Control collagen gels (n = 9) had a mean ± standard deviation diameter (mm) of 15.943 ± 0.308 for days 0–7 with no contraction observed. PMSC collagen gels (n = 9) (*p* values = Student *t*-test comparison of control collagen gels) versus PMSC gels for each day had a mean ± standard deviation diameter (mm) of 15.800 ± 0.201 for day 0 (*p* = 0.2632), 11.230 ± 0.590 (*p* < 0.0001) for day 3, 9.230 ± 0.448 (*p* < 0.0001) for day 5, and 7.460 ± 0.630 (*p* < 0.0001) for day 7. No significant difference was observed for day 0 with PMSCs seeded in gels, and each day after (days 3–7) displayed significant differences in average diameter when compared to control gels (see Figure 6). Additionally, each PMSC collagen gel was significantly different when compared to each other on 0–7 days (*p* < 0.0001).

**Figure 7:**
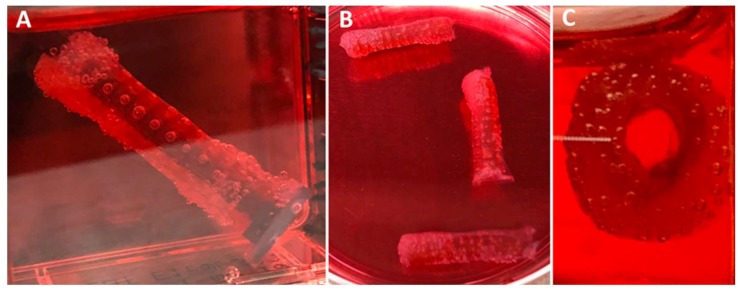
Images of 3D printed XL-PVA stents with maturation plug and collagen/PMSC at day 5 (**A**) and with plug removed (**B**,**C**).

**Figure 8:**
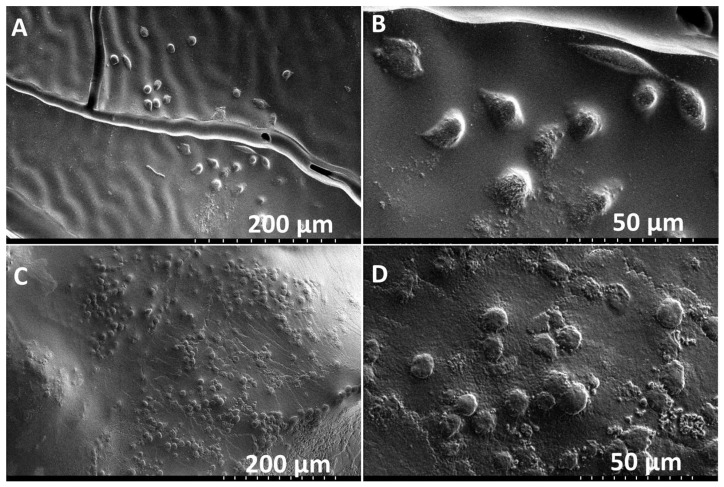
Cryo-SEM images of XL-PVA with cholangiocytes on the surface (**A**,**B**). Cryo-SEM images of collagen with cholangiocytes on the surface (**C**,**D**). (**A** and **C**, scale bar = 200 μm) and (**B** and **D**, scale bar = 50 μm).

Similarly, PMSC collagen contraction was observed around the XL-PVA stents over the course of 7 days (see Figure 7). Upon removal of the maturation plug from the PMSC collagen seeded stents, patency of the inner lumen was observed and maintained at day 5 and contraction continued to day 7 (see Figure 7C). CryoSEM images showed that both XL-PVA and collagen surfaces promoted cell attachment with cholangiocytes (see Figure 8).

**Figure 9:**
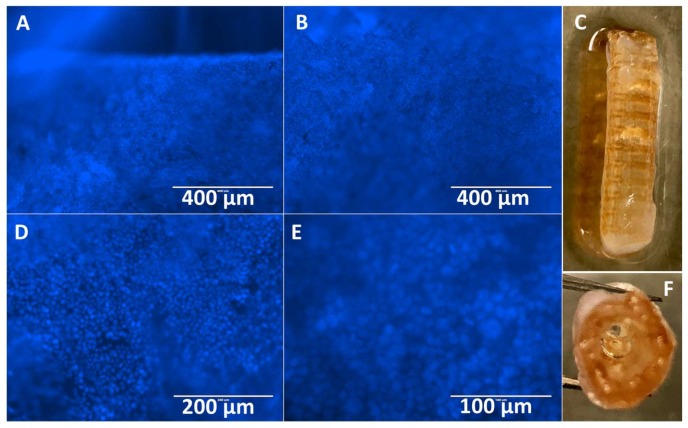
Images of Hoechst 33342 fluorescence (blue) staining of cholangiocytes on the outer stent surface (**A**,**B**), and image of outer stent (**C**). Images of Hoechst 33342 fluorescence (blue) staining of cholangiocytes on the inner stent surface (**D**,**E**) and image of inner lumen of stent (**F**) at day 7. (**A** and **B**, scale bar = 400 μm). (**D**, scale bar =200 μm). (**E**, scale bar = 100 μm).

**Figure 10:**
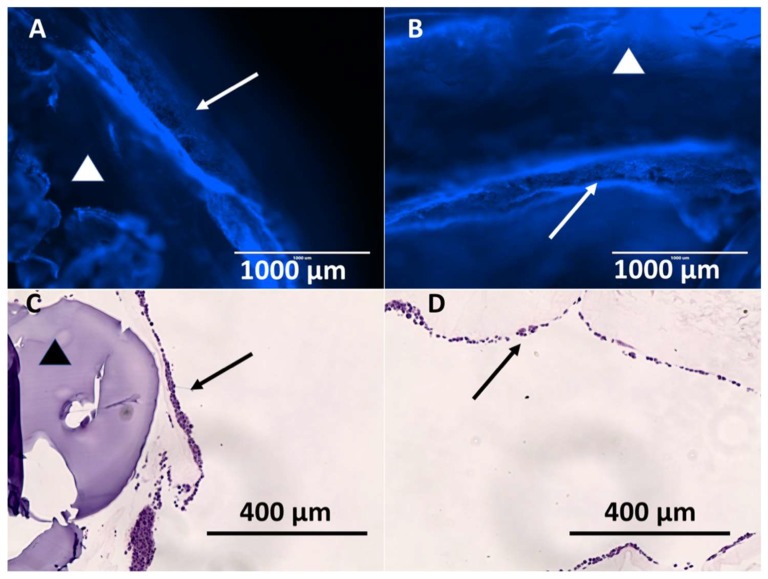
Images of Hoechst 33342 fluorescence (blue) staining of cholangiocytes on the outer stent surface (**A**) and of inner stent surface (**B**). (**A** and **B**, scale bar = 1000 μm). Images of H&E stain of outer stent surface (**C**) and of inner stent surface (**D**). (**C** and **D**, scale bar = 400 μm) (Arrows = cholangiocyte layer, and triangles = 3D printed XL-PVA area).

Stents containing XL-PVA, collagen, PMSC, and cholangiocytes that have matured for two weeks displayed tight uniform collagen coatings around the XL-PVA and densely bound monolayers of cholangiocytes on the surfaces (see Figure 9). Both inner and outer stent surface showed dense cholangiocyte surfaces. Additionally, cross-sections of inner and outer stent surfaces displayed contracted collagen with cholangiocyte monolayers visible by both fluorescent imaging and histology (see Figure 10).

## 4. Discussion

In this current proof-of-concept study, both a novel 3D printed biliary stent fabrication technique as well as a method for injection molding and coating these stents with a stem cell and collagen-cholangiocyte linings were reported. In these in vitro studies, predictable swelling of the hydrated stent matrix was achieved, with matrices absorbing nearly an equivalent water mass over the dehydrated cross-linked print. The mass of the stent can be manipulated through hydration, which may be important when deploying the dehydrated stent endoscopically into the wet environment of the common bile duct in vivo. These stents were successfully modified with barium contrast to create x-ray attenuation, an important factor for fluoroscopic visualization and localization during endoscopic procedures. A prior study of impregnated contrast materials showed barium to be more resilient than iodinated contrast materials when incorporated into the structure of 3D-printed constructs (i.e., surgical mesh used in that study) [22]. Additional PCL and other polymer coatings or drugs could be applied to PVA-based stents for a variety of material modifications including increased mechanical properties, enhanced cell attachment, and optimal drug delivery. Additionally, other polymer 3D print materials could be integrated with 3D printed injection molding systems.

The XL-PVA crosslinking method creates a stable and stiff 3D hydrogel with favorable collagen and cell adhesive properties. While the degree of crosslinking was not quantitively measured, it was qualitatively observed that shorter time periods of crosslinking and more solid prints resulted in more unstable stents that fractured randomly upon hydration due to uncross-linked regions. The overall size of the PVA print and macro-porosity in this study allowed for consistent and stable stent structures with good handling upon hydration. The 1 mm porosity allowed for consistent global crosslinking with suitable features for this method. 1 mm dimensions are also compatible with most consumer grade 3D printers. The PMSC-collagen gels in a 48-well configuration and in 3D printed stents showed progressive tonic contraction which contracts around and fuses with the print material. The PMSCs exhibit different adherence morphology in 2D and 3D cultures. PMSCs grown in collagen gels are able to form 3D arrangement and attachment within the collagen network. Cytoskeleton branching is able to form in all directions, which translates to global contraction of the collagen gels. This stem cell contraction is an important feature to consider when designing such collagen frameworks for tissue engineering, and such material combinations are not limited to hepatobiliary stents. A variety of vascular tissues and grafts may also be adapted using this technique with PMSCs as they are readily available non-invasive and non-controversial.

Cholangiocytes displayed similar adherence morphology in 2D and 3D cultures with a tightly bound single layer cobblestone pattern. The uniform cobblestone pattern occurred on both the tissue culture plastics and around the collagen stent as a coating. Both direct microscopic visualization along with fluorescent and histologic staining confirmed the presence of cholangiocytes on the surface of these 3D printed stents. It is hypothesized that the addition of an external cholangiocyte layer is intended to enhance integration and eventually fuse to the inner stent cholangiocyte layer. Biofilms generally occur on the surface of plastics which allow adherence and biofilm formation [15,16]. It is suggested that by using patient cells or other tightly bound cell types, a stent surface could be engineered with cells to reduce entrance and adherence of harmful bacteria. With such new collagen fabrication methods entering the market, such as recombinant human collagen, a very wide variety of biocompatible collagen 3D print-based technologies may be developed in the near future with patient cells [28,29].

Cumulatively, the parameters in this study are relevant for creating, testing and manipulating many 3D printed formats besides biliary stents. The design of this stent is customizable and can be custom-designed to match patient-specific anatomies from medical imaging. Coupled with cryogenic storage and on-demand subtractive sculpting, stents with variable sizes could be achieved through use of these methods [30,31,32,33]. Although the incorporation of cholangiocytes onto the 3D printed stent’s surface was successfully accomplished, additional work is anticipated, including long-term cell viability and optimal thickness, animal studies, and mechanical testing which will demonstrate how effectively these coatings and materials integrate and maintain stent patency.

## 5. Conclusions

In conclusion, this study demonstrated in vitro a proof-of-concept synthesis of 3D printed plastic biliary stents impregnated with barium along with a stem cell-collagen-cholangiocyte coating. The biodegradation of the PVA is an ongoing study where we are examining degradation in bile and animal models. We hope to publish future findings on the effects of crosslinking on the degradation of PVA in vitro and in vivo. It is hypothesized that less crosslinked materials will result in faster biodegradation and mechanical degradation. However, they may not be suitable for implantation and handling. Overall, a wide variety of compatible 3D printing materials could be used with this method and it is not limited to crosslinked PVA. To our knowledge, this is the first report of 3D printing technology being used to fabricate custom biliary bio-stents. Whereas the 3D printing allows for customization of the stent structure and impregnation of barium for visibility, the properties of the matrix allows for variations of the mass and swelling of the stent wall composition. These are further modified by the living coating, which promotes contraction and integration of the components of the stent. Aside from the cholangiocytes incorporated on the surface in the present study, the collagen coating process may facilitate other enhancements on the 3D printed biliary stent’s surface. All of these factors are in keeping with the novel capabilities of 3D printing to facilitate customizable, patient-specific medicine. These advancements may help facilitate custom biliary stent design, aimed at improving patency and patient care.

## 6. Patents

Alexander, J.S.; Boyer, C.J. 3D Printed Polyvinyl Alcohol Medical Devices and Methods of Activation. Assignee: LSU Health Sciences Center Shreveport, LA. U.S. Non-Provisional Patent Application 15/721,561. Wang, Y. Digested placental microvilli culture: a simple, efficient, and reproducible process to obtain stromal/mesenchymal stem (stem cell-like) cells from human term placenta. Assignee: LSU Health Sciences Center Shreveport, LA. U.S. Provisional Patent Application 62/658,084.
